# First description of the female subimago of *Behningia baei* McCafferty & Jacobus, 2006 (Ephemeroptera, Behningiidae), with notes on nymphal sand-burrowing behaviour

**DOI:** 10.3897/BDJ.14.e210906

**Published:** 2026-09-04

**Authors:** Sedtawut Kwanboon, Damrong Chainthong, Boonsatien Boonsoong

**Affiliations:** 1 Animal Systematics and Ecology Speciality Research Unit (ASESRU), Department of Zoology, Faculty of Science, Kasetsart University, Bangkok, Thailand Animal Systematics and Ecology Speciality Research Unit (ASESRU), Department of Zoology, Faculty of Science, Kasetsart University Bangkok Thailand; 2 Biodiversity Center Kasetsart University (BDCKU), Bangkok, Thailand Biodiversity Center Kasetsart University (BDCKU) Bangkok Thailand

**Keywords:** adult stage, behaviour, *
Behningia
*, burrowing mayfly, insect

## Abstract

**Background:**

*Behningia baei* McCafferty & Jacobus, 2006 was only known from the nymphal stage and recorded from two localities in Thailand. While the winged stages have been described for four congeners of *Behningia*, those of *B. baei* have not yet been documented.

**New information:**

Here, we present the first morphological description of the female subimago of *B. baei* based on reared specimens collected from sandy-bottomed rivers in northern Thailand. We compare diagnostic characters of the female subimago with those of known species of *Behningia*. In addition, we provide data on burrowing behaviour in fine-sand substrate. Our findings bring new biological and behavioural data for this rare genus.

## Introduction

Only five species of the genus *Behningia* Lestage, 1930 (Behningiidae) have been described worldwide. This genus is distributed in the Palearctic and Oriental Regions. Nymphs of *Behningia* are specialised burrowers that inhabit fine-sand substrates in rivers (Hubbard 1994, Zheng et al. 2024). The winged stages of *B. lestagei* Motas & Bacesco, 1937; *B. sushii* Zheng & Zhou, 2024; *B. tshernovae* Edmunds & Traver, 1959; and *B. ulmeri* Lestage, 1930 have previously been described (Tshernova 1938, Edmunds and Traver 1959, Keffermüller 1959, Hubbard 1994, Zheng et al. 2024). However, the winged stage of *Behningia baei* McCafferty & Jacobus, 2006 remains unknown. *Behningia baei* has been recorded only in the nymphal stage from Phitsanulok and Chiang Mai Provinces, Thailand (McCafferty and Jacobus 2006, Kwanboon and Boonsoong 2025). During field surveys for mayfly specimens in the Mae Chaem River, we collected mature nymphs of *B. baei* and successfully reared them to the female subimago under laboratory conditions. In addition, we documented larval burrowing behaviour under laboratory observation. These findings provide new morphological and biological information for *B. baei* and contribute to a better understanding of the taxonomy and biology of Behningiidae.

## Materials and methods

### Specimen collecting

**Figure 1.**
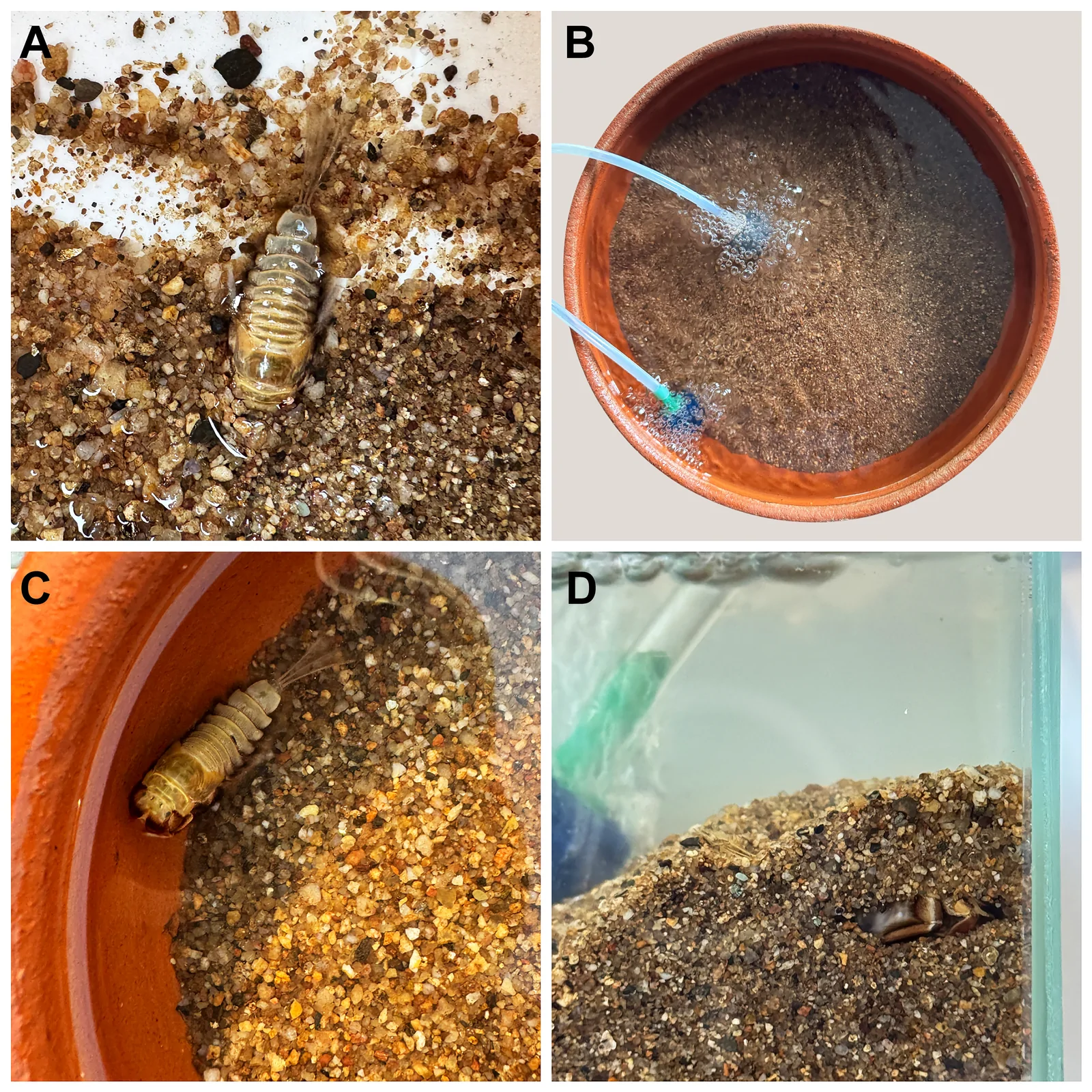
**A** Nymph of *Behningia baei*; **B** Rearing chamber (earthen pot); **C** Nymph acclimatisation; **D** Observation of sand-burrowing behaviour in an aquarium glass tank.

The nymphs of *B. baei* were collected with a D-framed net in fine-sandy habitat, the Mae Chaem River, Chiang Mai Province, Thailand (Fig. 1A). Nymphs were reared in an earthenware pot (30 cm diameter), which helped reduce the water temperature. The rearing chamber was filled with fine sandy substrate collected from the locality and connected to an air supply via aquarium tubing (Fig. 1B). Nymphal exuviae and subimago specimens were preserved in absolute ethanol.

### Morphology observation

Measurements and photographs were taken using a stereomicroscope (Nikon SMZ800 and ZEISS Stemi 305). For scanning electron microscopy (SEM), egg specimens were dried in a critical point dryer (CPD7501) and coated with gold using a sputter coater (SC7620). The specimens were observed and photographed with a FEI Quanta 450 SEM. The final plates were prepared with Adobe Photoshop CC 2022. The material was deposited in the collection of the Zoological Museum at Kasetsart University in Bangkok, Thailand (ZMKU).

### Molecular analysis

A specimen of *Behningia baei* nymph was dissected from thoracic muscle for DNA extraction. Total DNA was extracted using a genomic DNA purification kit (NucleoSpin, Macherey-Nagel, Germany) according to the manufacturer’s protocol. The COI gene amplification was performed using LCO1490 and HCO2198 (Folmer et al. 1994). The polymerase chain reaction (PCR) conditions and procedure were as described by Kwanboon et al. (2021). The PCR products were sent to U2Bio Co., Ltd (Korea) for purification and sequencing. Other analysed sequences were obtained from GenBank: *Protobehningia merga* (MW792224); *Paradolania nujiangensis* (OQ439817); and *Dolania americana* (JQ662655). The genetic distances between the species were determined using the Kimura 2-parameter (K2P) distance (Kimura 1980) and calculated with the MEGA11 programme (Tamura et al. 2021). Sequence alignment and editing were performed using ClustalW in MEGA11.

### Behavioural observation

Nymphs of *Behningia baei* were collected from the Mae Chaem River and transported to the laboratory. The specimens (*n* = 4) were acclimatised in a rearing chamber (earthen pot) containing river sand to simulate their natural burrowing habitat (Fig. 1C). After acclimatisation, their behaviour was continuously recorded using a video camera in an aquarium glass tank (Fig. 1D). The recordings were subsequently examined to document burrowing behaviour, locomotion and other observable activities.

### Ethics statement

This research was approved by the Institutional Animal Care and Use Committee (IACUC), Faculty of Science, Kasetsart University, under Project No. ACKU68-SCI-016. All procedures followed institutional ethical guidelines.

## Taxon treatments

### Behningia baei

McCafferty & Jacobus, 2006

B4EA5848-6516-565B-B301-D6C44DE0D359

*Behningia baei* - McCafferty and Jacobus (2006): 47 (original description).

#### Materials

- **Type status:** Other material. **Occurrence:** catalogNumber: ZMKU Ephe-050; sex: 1 female; lifeStage: subimago and exuvium, reared from a mature nymph; occurrenceID: 638D3014-1DCE-5B45-B39C-64DC6C450FB2; **Taxon:** scientificName: *Behningia baei*; kingdom: Animalia; phylum: Arthropoda; class: Insecta; order: Ephemeroptera; family: Behningiidae; genus: *Behningia*; specificEpithet: *baei*; scientificNameAuthorship: McCafferty & Jacobus, 2006; **Location:** country: Thailand; countryCode: TH; stateProvince: Chiang Mai; locality: Mae Chaem District, Mae Chaem River; verbatimElevation: 470; verbatimCoordinates: 18°29'31"N 98°21'49"E; decimalLatitude: 18.51304; decimalLongitude: 98.35690; **Identification:** identifiedBy: Boonsatien Boonsoong; Sedtawut Kwanboon; **Event:** samplingProtocol: aquatic net; eventDate: 29 Mar 2025; **Record Level:** institutionCode: Zoological Museum Kasetsart University (ZMKU); collectionCode: ZMKU Ephe; basisOfRecord: PreservedSpecimen; **Material Entity:** preparations: whole animal (EtOH)

#### Description

**Figure 2.**
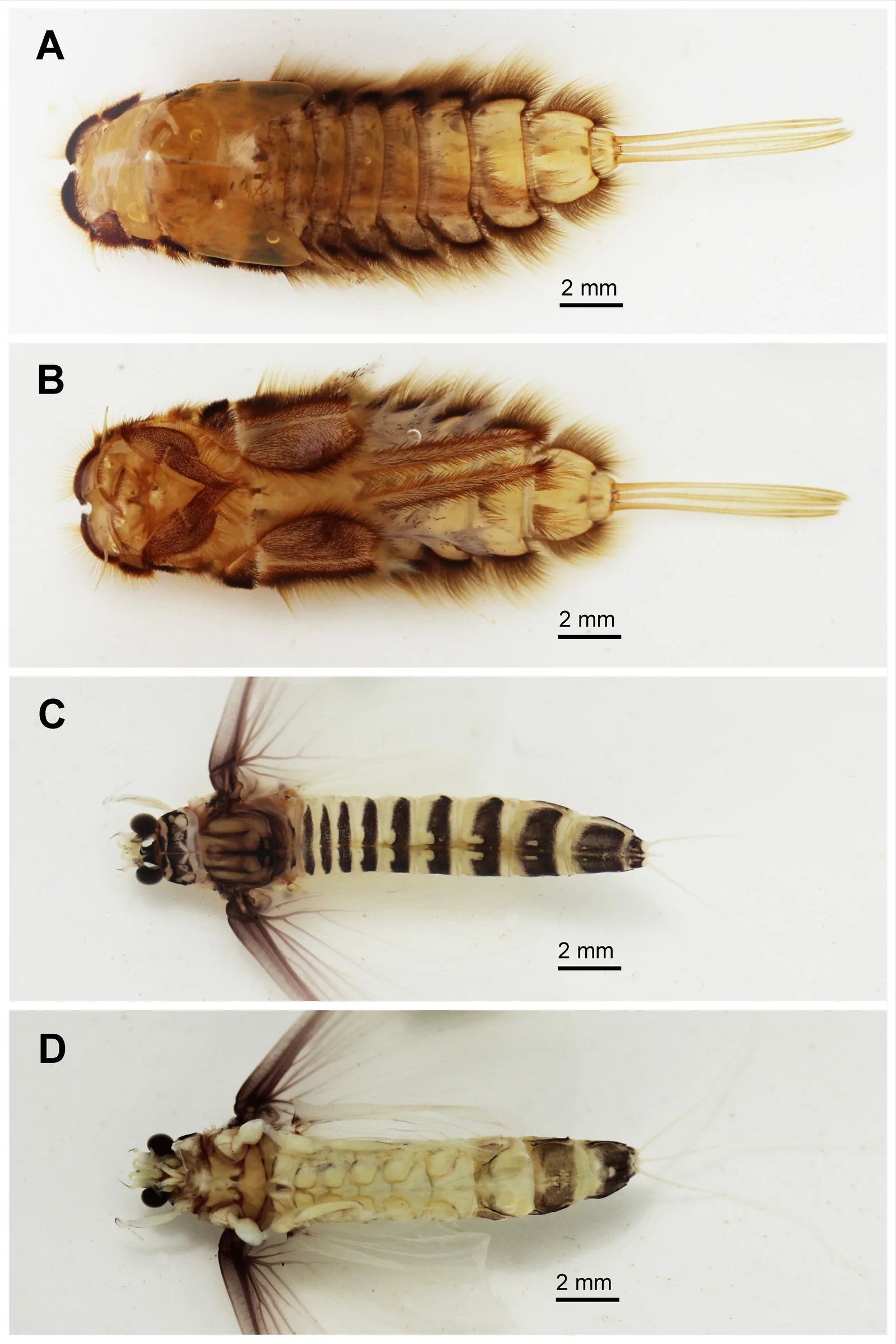
Habitus of *Behningia baei*. **A** Nymphal exuvium (dorsal view); **B** Nymphal exuvium (ventral view); **C** Female subimago (dorsal view); **D** Female subimago (ventral view).

**Female subimago (in alcohol)**: Body length 21.6 mm, excluding caudal filament (Fig. 2C and D).

**Figure 3.**
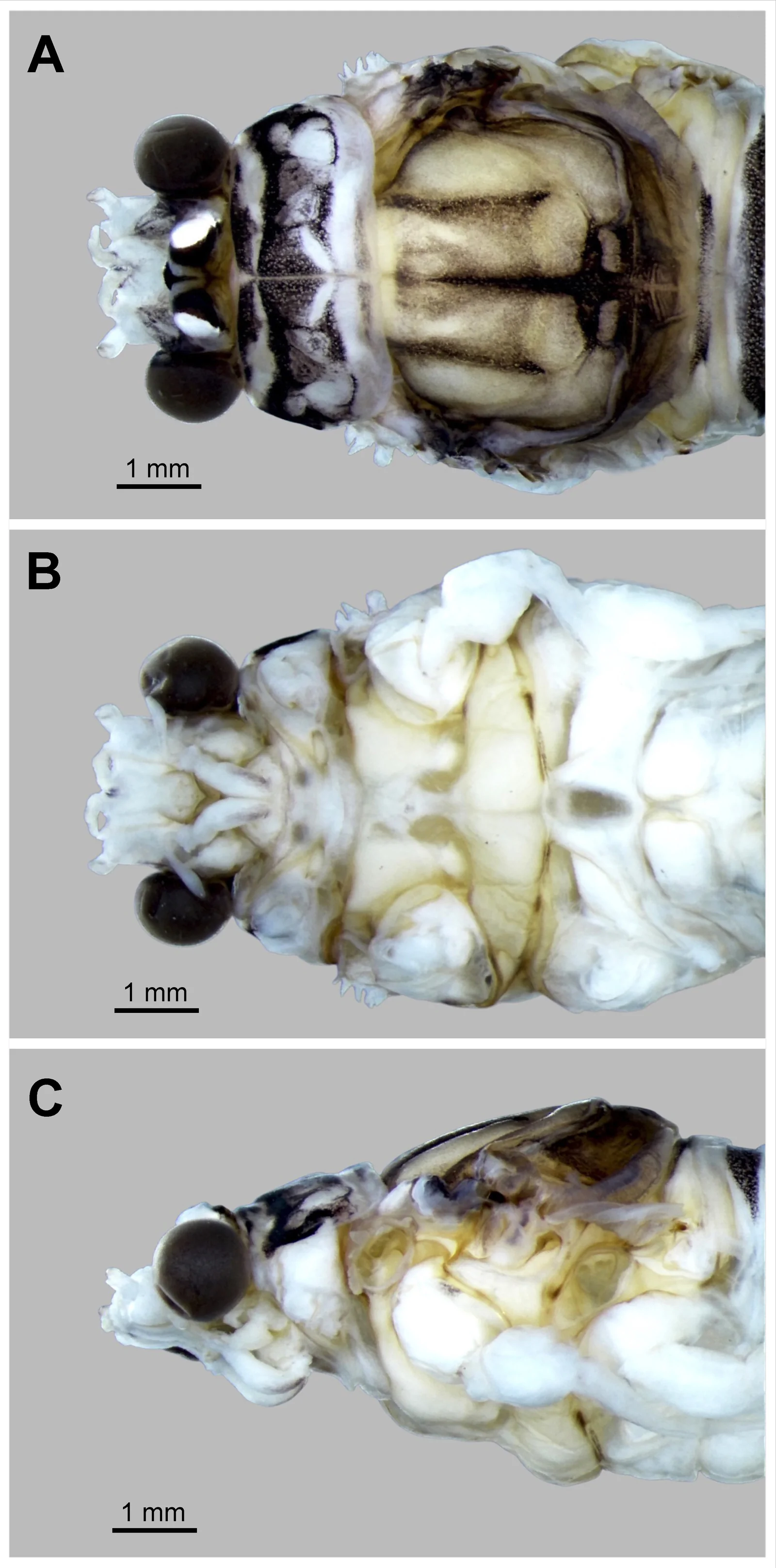
Head and thorax of *Behningia baei*. **A** Dorsal view; **B** Ventral view; **C** Lateral view.

**Head**: Head whitish with dark colour pattern (Fig. 3A–C). Black compound eye on dorsolateral margin, globular in shape; three ocelli located between compound eyes, median ocellus smallest and black, two lateral ocelli white, inner margin of ocelli black. Scape and pedicel whitish, flagella greyish. Vestigial mouthparts present posteriorly on ventral side of head.

**Figure 4.**
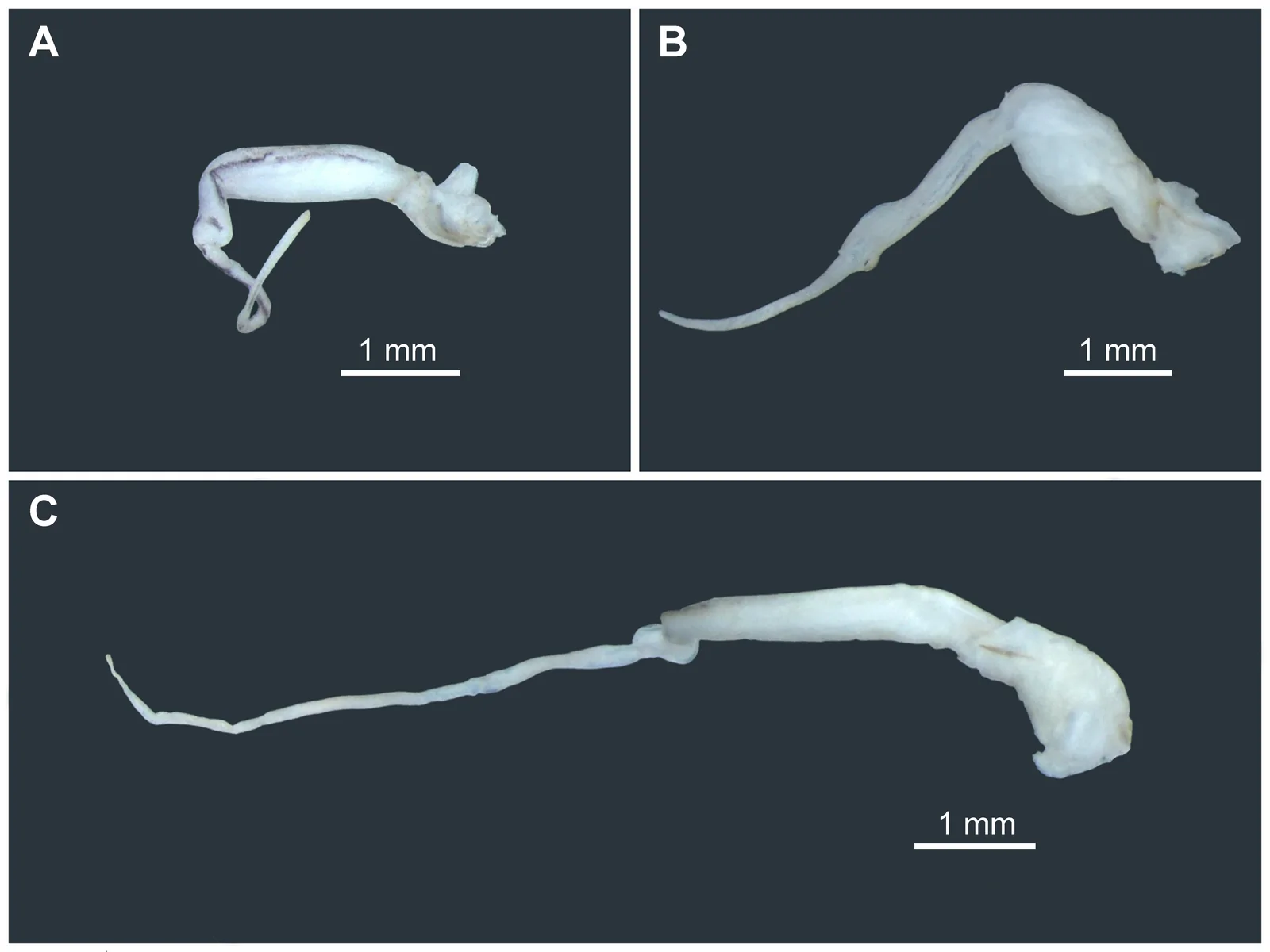
*Behningia baei*, female subimago morphology. **A** Fore-leg; **B** Middle leg; **C** Hind-leg.

**Figure 5.**
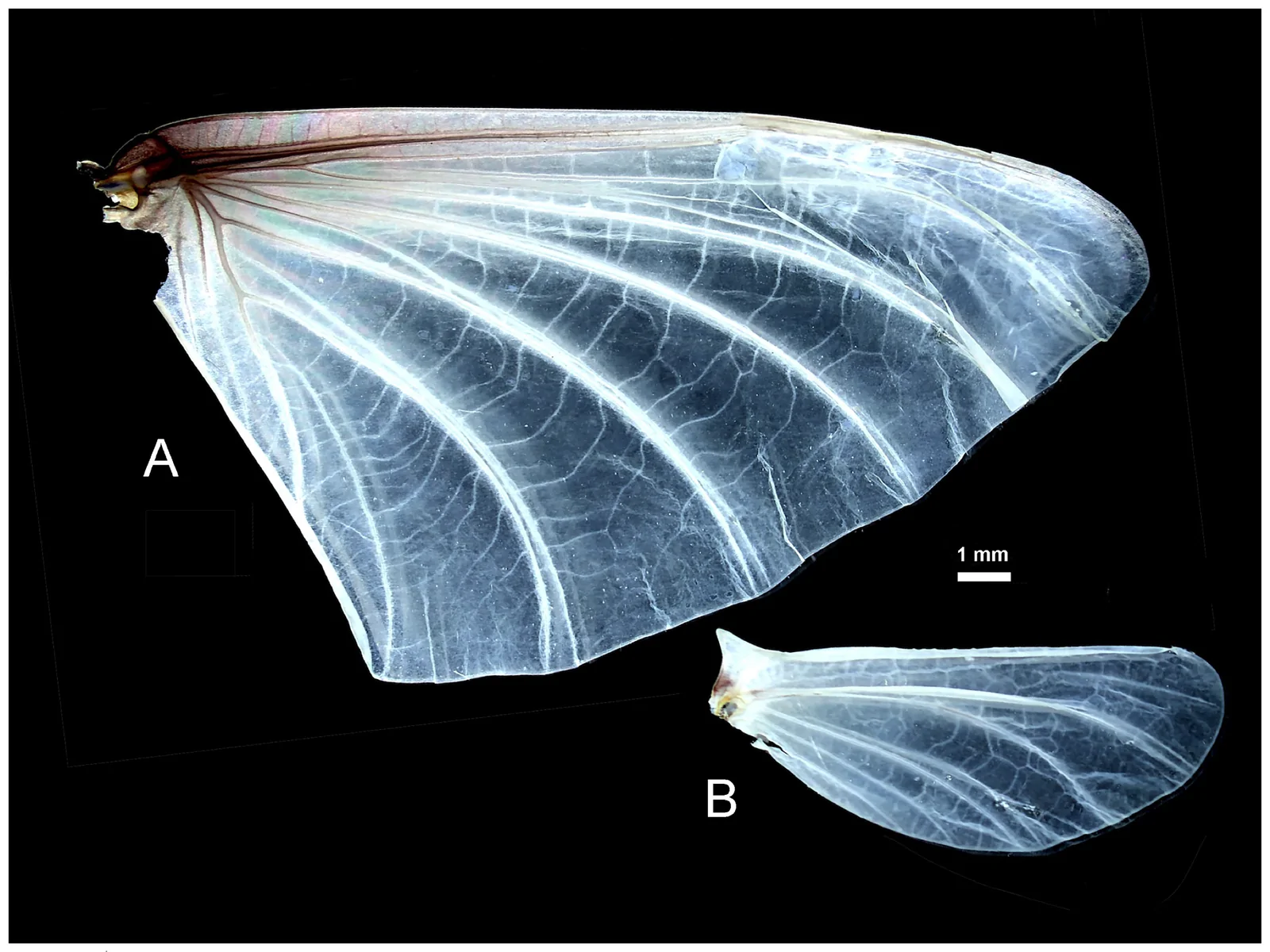
*Behningia baei*, female subimago morphology. **A** Fore-wing; **B** Hind-wing.

**Thorax**: Pronotum width slightly equal to head width, length: width ca. 1: 2.7, colour dark brown with white marking, mesonotum dark brownish (Fig. 3A). Fore-leg: poorly developed and twisted, white in colour, tibia and tarsus fused and slender (Fig. 4A). Mid-leg: whitish, claw absent, femur stout, tibia twisted, length ratio of femur: tibia: tarsus ca. 1: 1.4: 1.4 (Fig. 4B). Hind-leg: longest, white in colour, claw absent, glabrous tibia fused with tarsus (Fig. 4C). Fore-wing: greyish translucent ca. 19.6 mm, dark brownish at base. Ten longitudinal veins present in five pairs: RSa2+iRS, RSp+MA1, iMA+MA2, MP1+iMP and MP2+CuA1. Fork of CuA section with one intercalary, intercalary reticulations along hind margin of fore-wing (Fig. 5A). Hind-wing: sharp costal projection, greyish translucent, with dark brown dot at base, venation as in Fig. 5B.

**Figure 6.**
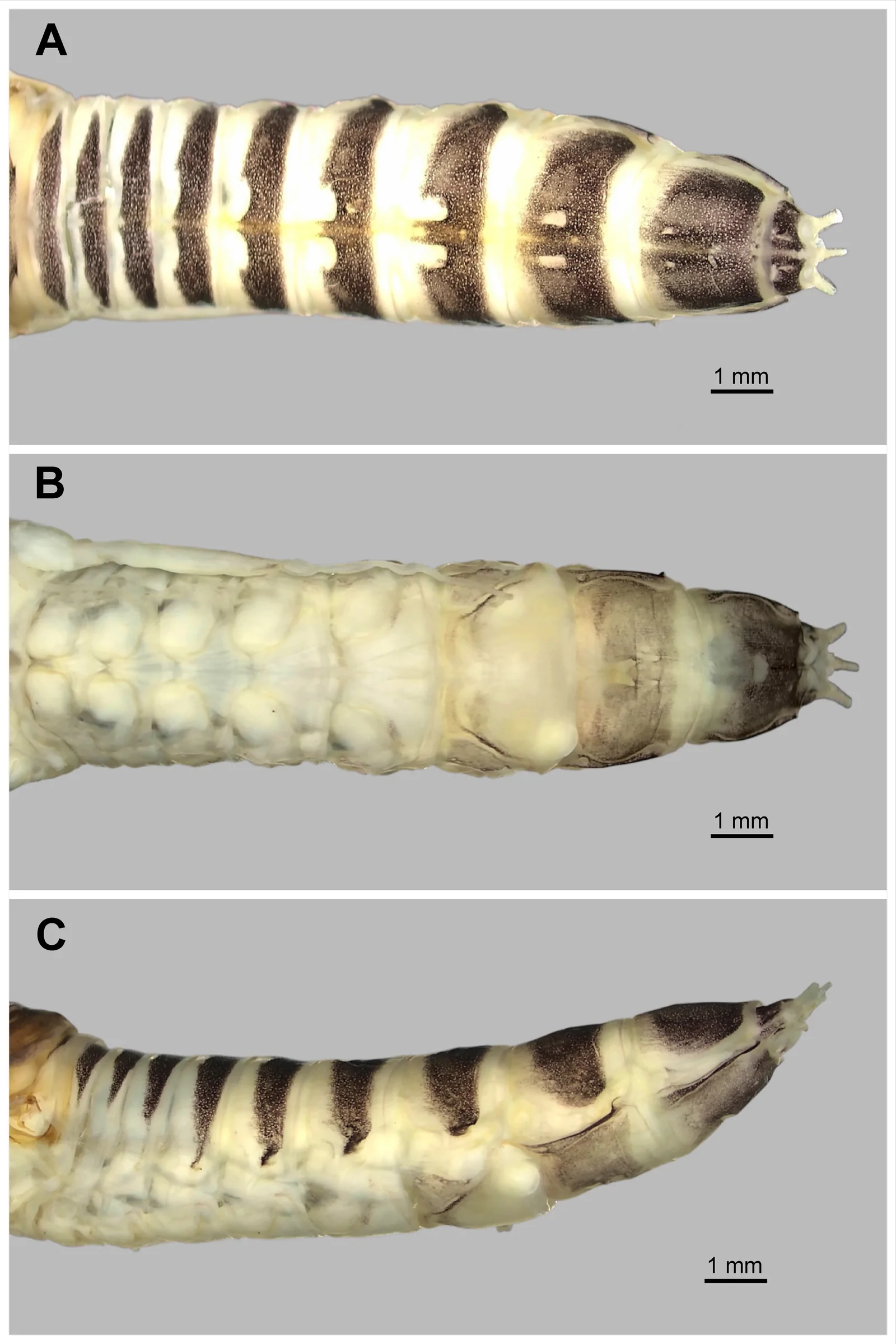
*Behningia baei*, abdomen of female subimago. **A** Dorsal view; **B** Ventral view; **C** Lateral view.

**Abdomen**: Abdominal terga whitish, with dark brown to black band markings and a pair of whitish median spots on each abdominal tergum (Fig. 6A). Abdominal sterna I–VII (Fig. 6B), brownish on sterna VIII–X; abdominal segment IX narrowed posteriorly; abdominal sterna I–VII with a pair of oblique ridges on each segment (Fig. 6C). Subgenital plate (sternum VII) broad, subtrapezoidal, slightly wider than long (Fig. 6B and Fig. 9B). Three caudal filaments present, translucent white.

**Figure 7.**
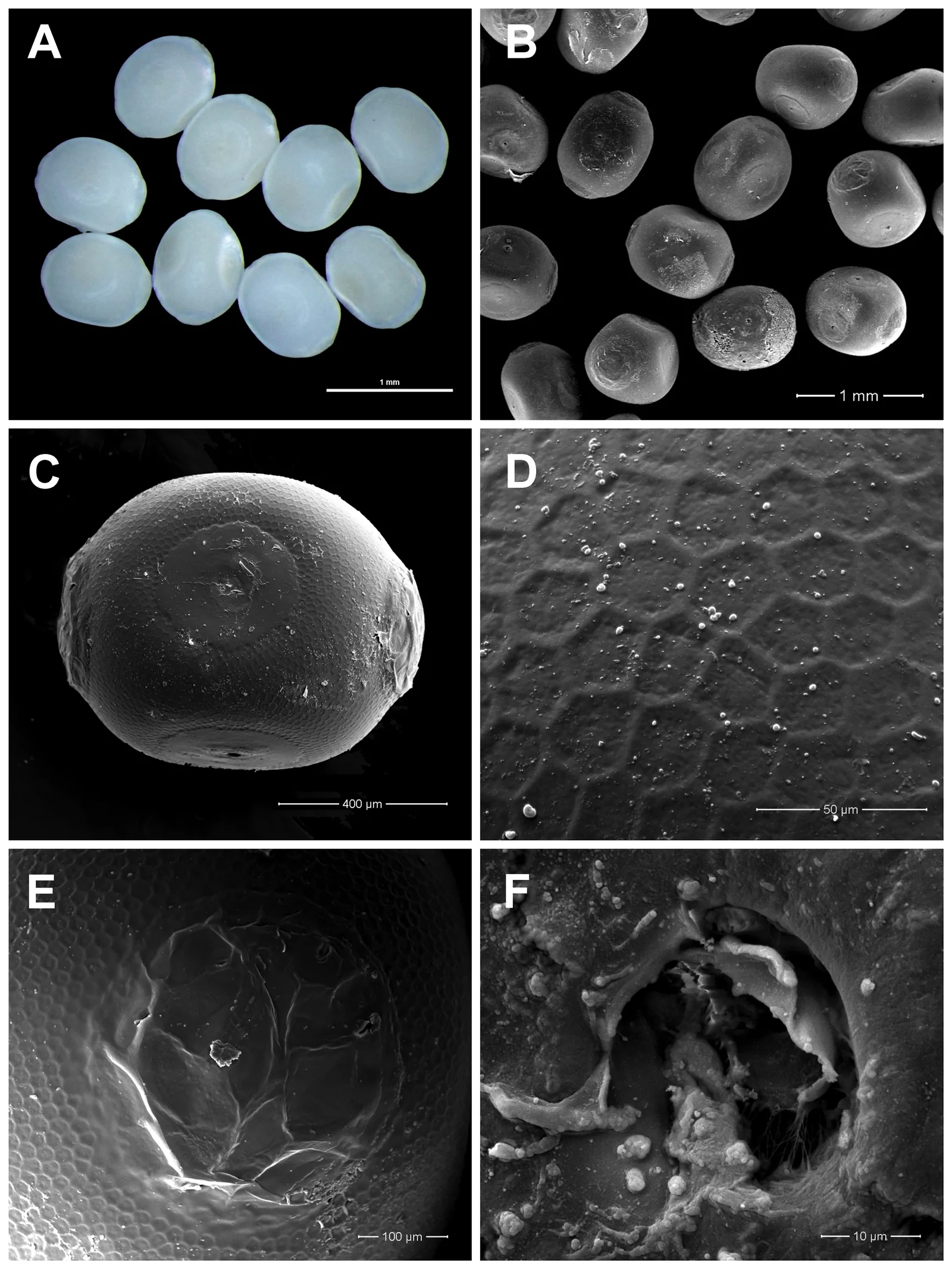
*Behningia baei*, egg morphology. **A** General outline (Light Microscope); **B** General outline (Scanning Electron Microscope); **C** General outline of egg lateral view; **D** Chorion surface; **E** Polar cap; **F** Micropyle.

**Egg**: Dissected from female subimago; length 875–968 μm, width 655–729 μm (*n* = 9); oval-shaped, slightly longer than wide (Fig. 7A–C); polar attachment areas smooth and circular, thin adhesive layer, one at each pole and several along the equatorial zone (Fig. 7C); Chorion distinctly reticulate, consisting of irregular polygonal meshes delimited by low, sinuous ridges, giving the chorion a honeycomb-like appearance, surface between the ridges smooth (Fig. 7D); funnel-form micropyle in the centre of circular accumulations of adhesive material only at the equatorial zone (Fig. 7E).

#### Diagnosis

The female subimago of *Behningia baei* can be separated from those of known female subimago of *Behingia* species, based on the following characteristics: i) one intercalary between CuA section (Fig. 5A); ii) reticulations along hind margin of fore-wing (Fig. 5A); iii) glabrous hind tibiae (Fig. 4C); iv) subgenital plate subtrapezoidal, slightly wider than long (Fig. 6B) and v) dark brown colour at base of fore-wing (Fig. 5A).

#### Distribution

Thailand (Phitsanulok and Chiang Mai Provinces).

#### Ecology

This species was found in fine sandy substrate. The nymphs of *B. baei* were collected from the same river as *Protobehningia merga* (Mae Chaem River) (Kwanboon et al. 2021).

#### Conservation

The occurrence of *B. baei* together with *P. merga* in a river, characterised by fine sandy substrate, highlights the importance of this habitat for behningiid mayflies. As these insects appear to be closely associated with this specialised microhabitat, changes in substrate composition may adversely affect their populations. However, additional ecological studies are needed to evaluate the extent of this habitat dependence and its implications for conservation.

#### Rearing

Two nymphs were collected from the locality on 29 March 2025. Only one survived and successfully moulted on 9 April 2025. During the rearing period, the water temperature was approximately 29°C. The mature nymph emerged as the female subimago on 30 May 2025, but subsequently fell into the water. The nymphal exuviae (Fig. 2A and B) and female subimago (Fig. 2C and D) were preserved in absolute ethanol.

#### Burrowing behaviour 

**Figure 8.**
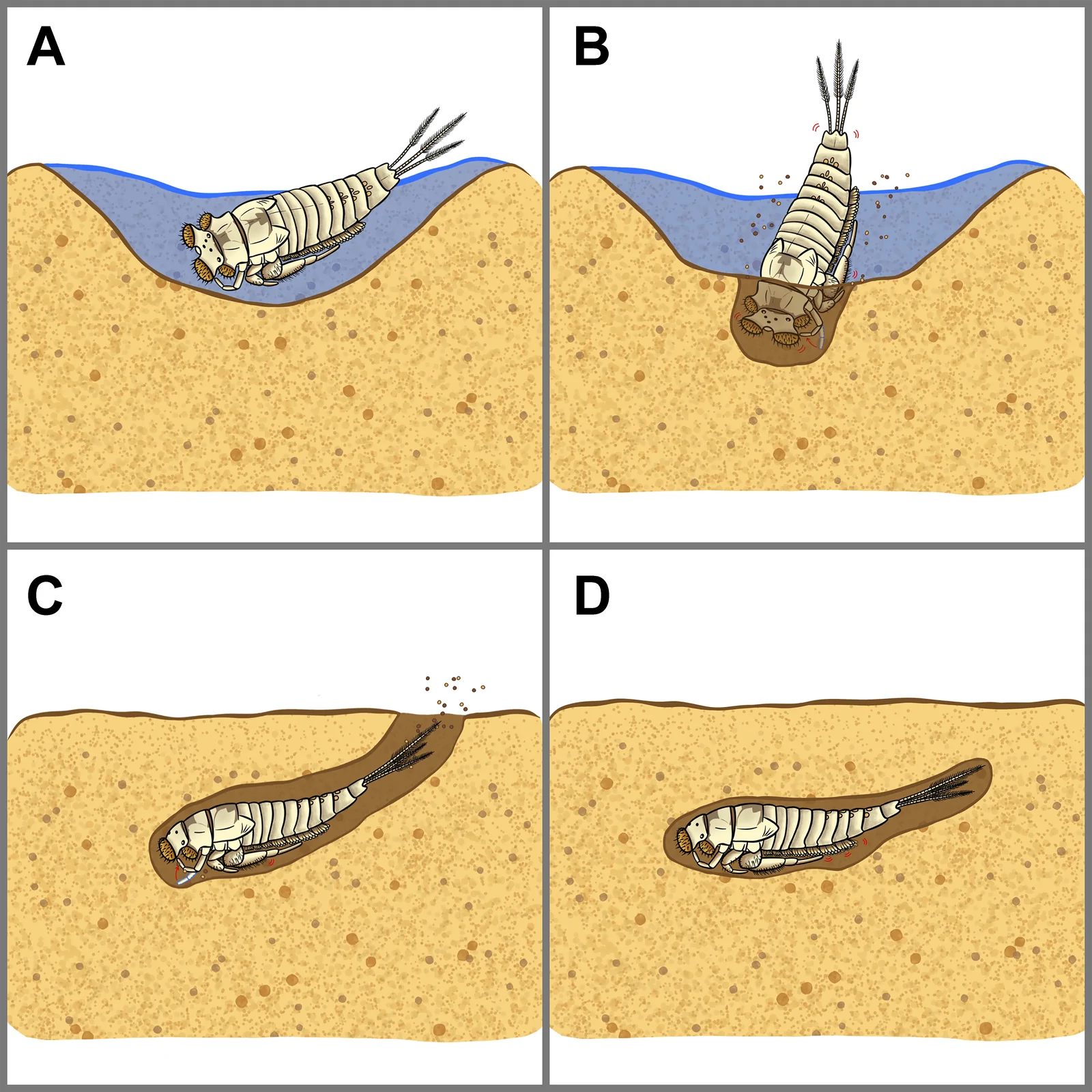
Stages of sand-burrowing behaviour of *Behningia baei* nymph. **A** pre-burrowing; **B** Head burrowing; **C** Body burrowing; **D** Chamber construction with gill ventilation.

**Figure 9.**
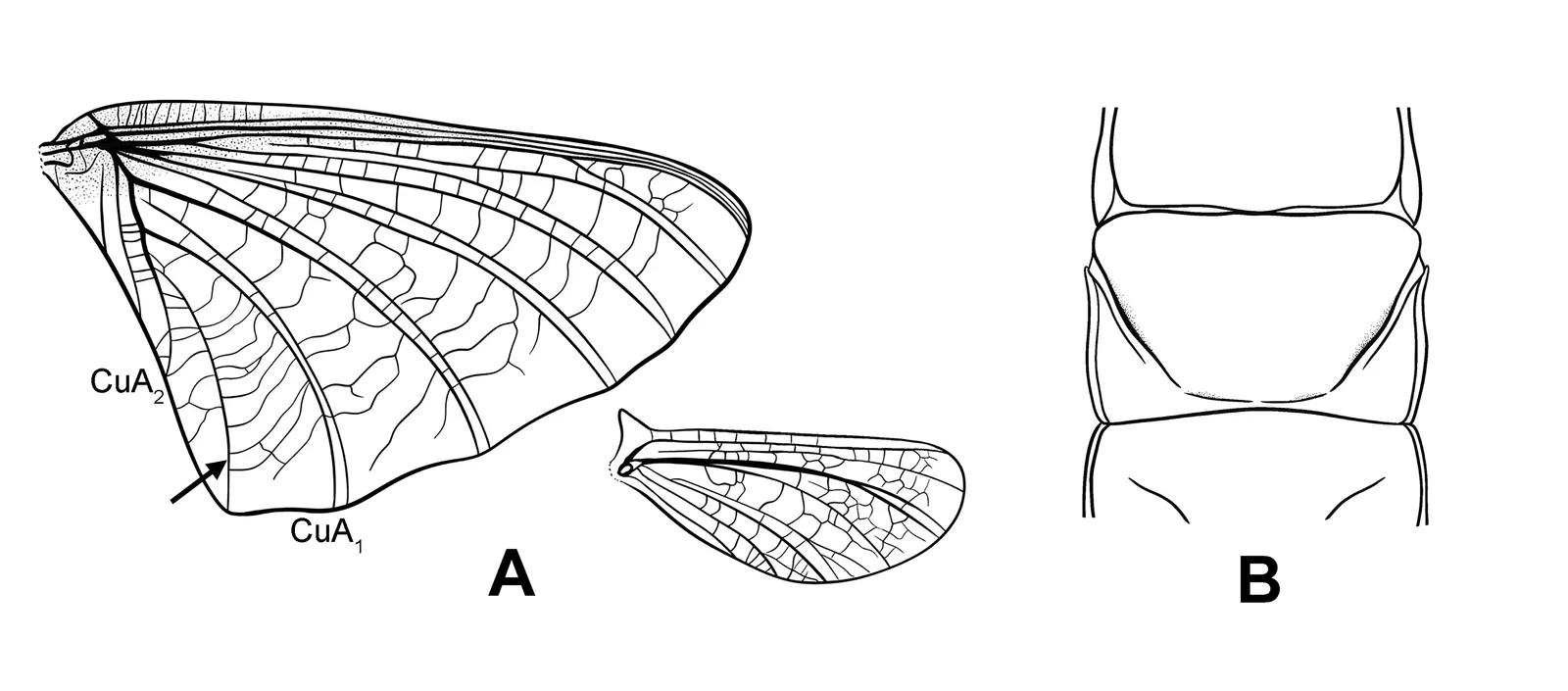
Female subimago *Behningia baei*. **A** Fore-wing and hind-wing; **B** Genital plate.

The burrowing behaviour of the nymph of *B. baei* was divided into four stages: i) pre-burrowing; ii) head burrowing; iii) body burrowing and iv) chamber construction with gill ventilation. During the pre-burrowing stage, the nymph swam close to the sandy substrate, while maintaining its body parallel to the substrate surface (Fig. 8A). After selecting a suitable location, it remained briefly motionless before initiating burrowing. Burrowing began by inserting the head into the sandy substrate at a steep angle to the substrate surface. The head served as the primary structure for burrowing, while the fore-legs helped loosen and transport sand particles. As burrowing progressed, the anterior part of the body gradually moved beneath the substrate, while the posterior part remained above the surface (Fig. 8B). Following head burrowing, the larva continued to advance deeper into the sandy substrate. The legs became the primary structures for burrowing, enabling the larva to advance deeper until the entire body was beneath the substrate. As burrowing progressed, the body remained almost parallel to the substrate surface, with only the posterior end of the abdomen and the abdominal gills remaining close to the substrate surface (Fig. 8C). After the entire body was beneath the sandy substrate, the larva excavated a small chamber through coordinated leg movements (Fig. 8D). Rhythmic movements of the ventral abdominal gills generated a continuous flow of water through the chamber. A permanent burrow was not maintained. The larva occupied a temporary chamber supported by its body and legs while remaining stationary. The chamber collapsed after the larva moved to another position beneath the substrate.

#### Molecular analysis

A partial mitochondrial cytochrome c oxidase subunit I (COI) gene sequence (658 bp) of *B. baei* was generated in the present study. This sequence was analysed together with available COI sequences of *Paradolania nujiangensis*, *Protobehningia merga* and *Dolania. americana* retrieved from GenBank to evaluate intergeneric genetic divergence within Behningiidae (Table 1). The Kimura 2-parameter (K2P) model amongst the four genera of Behningiidae ranged from 19.0% to 23.7%. The lowest genetic distance was observed between *P. nujiangensis* and *D. americana* (19.0%), followed by *B. baei* and *P. merga* (21.6%), *P. merga* and *D. americana* (22.4%), *P. nujiangensis* and *B. baei* (22.6%) and *P. nujiangensis* and *P. merga* (23.4%), whereas the highest genetic distance was found between *B. baei* and *D. americana* (23.7%).

## Identification Keys

### Key to known *Behningia* species of adults

**Table:** 

1	One intercalary vein in fork of fore-wing CuA (Fig. 9A)	2
–	Two intercalary veins in fork of fore-wing CuA (see Peters and Gillies (1991), figs. 12 and 13)	*B. ulmeri* Lestage, 1930
2	Intercalary reticulations along hind margin of fore-wing	3
–	No intercalary reticulations along hind margin of fore-wing (see Edmunds and Traver (1959), figs. 24 and 25)	*B. tshernovae* Edmunds & Traver, 1959
3	Hind-leg without setae	4
–	Hind-leg with setae (see Keffermüller (1959), fig. 2c and Plate V)	*B. lestagei* Motas & Bacesco, 1937
4	Fore-wing without colour; subgenital plate subtriangular (see Zheng et al. (2024), fig. 10B)	*B. sushii* Zheng & Zhou, 2024
–	Fore-wing with colour; subgenital plate subtrapezoidal (Fig. 9B)	*B. baei* McCafferty & Jacobus, 2006

(portions adapted from Hubbard (1994))

## Discussion

During field surveys for *Behningia baei* in the Mae Chaem River, we successfully collected only seven nymphs. Early-instar nymphs in December 2024 were about 5-7 mm (Kwanboon and Boonsoong 2025), whereas in March 2025, they were about 20-25 mm. In this study, a mature nymph emerged in May. This species possibly emerges in the early rainy season, when it lays eggs during flooding to ensure the survival of hatched nymphs.

Successful laboratory rearing of *Behningia baei* from nymph to female subimago enabled the first description of this species. Four species of the genus *Behningia* have been described in winged stages: *B. ulmeri*, *B. tshernovae*, *B. lestagei* and *B. sushii* (Tshernova 1938, Edmunds and Traver 1959, Keffermüller 1959, Hubbard 1994, Zheng et al. 2024). In this study, the morphology of *B. baei* reveals that it is closely related to *B. sushii* from China, as both share characteristics, such as a single intercalary between the CuA section and glabrous legs. However, the two species differ in the colouration at the base of the fore-wing, which is dark brown in *B. baei*, but translucent in *B. sushii*, with different patterns on abdominal terga and the shape of the subgenital plate (Zheng et al. 2024). Nevertheless, the overall morphological similarity between the two species is consistent with their shared distribution in the Oriental Region. The three species known from the Palaearctic Region can also be distinguished from *B. baei* by several morphological characters. *Behningia lestagei* is distinguished from *B. baei* by its densely setose legs. In addition, *B. ulmeri* possesses two intercalary veins between the CuA section, whereas *B. tshernovae* lacks the reticulate veinlets along the hind margin of the fore-wing (Tshernova 1938, Edmunds and Traver 1959, Keffermüller 1959, Hubbard 1994).

The burrowing behaviour observed in *B. baei* closely resembles that reported for *Dolania americana* by McCafferty (1975). In both species, the larvae burrow directly into loose sandy substrate and subsequently maintain a temporary chamber beneath the body, while the gills beat continuously for ventilation (McCafferty 1975). Although the overall burrowing behaviour is similar, the present study provides a more detailed description of the behavioural sequence, recognising four successive stages (pre-burrowing, head burrowing, body burrowing and chamber construction with gill ventilation).

The intergeneric COI genetic distances amongst the four genera of Behningiidae ranged from 19.0% to 23.7%. These values are comparable to those reported for the closely-related burrowing mayfly (Potamanthidae), in which intergeneric genetic distances ranged from 17.1% to 21.2% using the Kimura 2-parameter model (Kwanboon et al. 2021). This similarity suggests that the level of genetic divergence amongst behningiid mayfly genera is consistent with that observed in other burrowing mayfly lineages.

Biological information on Behningiidae remains limited compared with studies of its morphology and taxonomy. Although the size distribution of *Dolania americana* has been extensively investigated (Tsui and Hubbard 1979, Harvey et al. 1980, Hubbard 1994), comparable information is available for only a few Asian species (Park et al. 2019, Kwanboon et al. 2021, Zheng et al. 2024). Consequently, life history traits and behavioural adaptations of most behningiid mayflies remain poorly documented.

## Supplementary Material

XML Treatment for Behningia baei

## Figures and Tables

**Table 1.** Genetic distances (COI) between genera of Behningiidae using the Kimura 2-parameter.

**Species (accession number)**		**1**	**2**	**3**	**4**
**1**	*Behningia baei* (PZ686065)				
**2**	*Paradolania nujiangensis* (OQ439817)	22.60			
**3**	*Dolania americana* (JQ662655)	23.70	19.00		
**4**	*Protobehningia merga* (MW792224)	21.60	23.40	22.40	
